# Where do knowledge-intensive firms locate in Germany?—An explanatory framework using exponential random graph modeling

**DOI:** 10.1007/s10037-023-00183-8

**Published:** 2023-02-28

**Authors:** Mathias Heidinger, Fabian Wenner, Sebastian Sager, Paul Sussmann, Alain Thierstein

**Affiliations:** Technische Universität München, Arcisstraße 21, 80333 Munich, Germany

**Keywords:** Locational choice, Firm networks, Transport infrastructure, Knowledge economy

## Abstract

This paper analyzes how positional and relational data in 186 regions of Germany influence the location choices of knowledge-based firms. Where firms locate depends on specific local and interconnected resources, which are unevenly distributed in space. This paper presents an innovative way to study such firm location decisions through network analysis that relates exponential random graph modeling (ERGM) to the interlocking network model (INM). By combining attribute and relational data into a comprehensive dataset, we capture both the spatial point characteristics and the relationships between locations. Our approach departs from the general description of individual location decisions in cities and puts extensive networks of knowledge-intensive firms at the center of inquiry. This method can therefore be used to investigate the individual importance of accessibility and supra-local connectivity in firm networks. We use attributional data for transport (rail, air), universities, and population, each on a functional regional level; we use relational data for travel time (rail, road, air) and frequency of relations (rail, air) between two regions. The 186 functional regions are assigned to a three-level grade of urbanization, while knowledge-intensive economic activities are grouped into four knowledge bases. This research is vital to understand further the network structure under which firms choose locations. The results indicate that spatial features, such as the population of or universities in a region, seem to be favorable but also reveal distinct differences, i.e., the proximity to transport infrastructure and different valuations for accessibility for each knowledge base.

## Abstract (de)

In diesem Beitrag wird analysiert, wie Positions- und relationale Daten in 186 Regionen Deutschlands die Standortentscheidungen wissensbasierter Unternehmen beeinflussen. Es wird ein innovativer Weg zur Untersuchung solcher Standortentscheidungen von Unternehmen durch Netzwerkanalyse vorgestellt, der exponential random graph modeling (ERGM) mit dem Interlocking Network Model (INM) verbindet. Durch die Kombination von Attribut- und relationalen Daten in einem umfassenden Datensatz erfassen wir sowohl die räumlichen Punkteigenschaften als auch die Beziehungen zwischen den Standorten. Unser Ansatz weicht von der allgemeinen Beschreibung individueller Standortentscheidungen in Städten ab und stellt umfangreiche Netzwerke von wissensintensiven Unternehmen in den Mittelpunkt der Untersuchung. Mit dieser Methode kann daher die individuelle Bedeutung von Erreichbarkeit und überörtlicher Konnektivität in Firmennetzwerken untersucht werden. Wir verwenden attributive Daten für Verkehr (Bahn, Flugzeug), Universitäten und Bevölkerung, jeweils auf funktionaler regionaler Ebene; wir verwenden relationale Daten für Reisezeit (Bahn, Straße, Flugzeug) und Häufigkeit von Beziehungen (Bahn, Flugzeug) zwischen zwei Regionen. Die 186 funktionalen Regionen werden einem dreistufigen Urbanisierungsgrad zugeordnet, während die wissensintensiven Wirtschaftstätigkeiten in vier Wissensbasen gruppiert werden. Die Ergebnisse zeigen, dass räumliche Merkmale wie die Bevölkerungszahl oder die Anzahl der Universitäten in einer Region günstig zu sein scheinen, aber auch deutliche Unterschiede aufweisen, z. B. die Nähe zur Verkehrsinfrastruktur und die unterschiedliche Bewertung der Erreichbarkeit für jede Wissensbasis.

## Introduction

Knowledge-based firms have been recognized as important drivers of urban development that increasingly shape and concentrate economic activity in only a few places (e.g., Friedmann 1986; Sassen 2001; Hall and Pain 2006; Taylor and Derudder 2016; Waiengnier et al. 2020). Transnational corporations depend on these knowledge-based firms, which function as central actors in knowledge creation and diffusion (Bathelt et al. 2004). This process has led to the formation of multilocational, often multinational, firm networks of knowledge-intensive firms offering their services globally (Derudder and Parnreiter 2014). Here, firms access heavily localized knowledge and make it available globally (Bathelt and Glückler 2011). Therefore, cities increasingly act as nodes in global flows of knowledge, capital, and goods in such networks of firm locations (Castells 1996; Sassen 2001; Smętkowski et al. 2021). Still, how well a city or region is positioned in the global economy depends on both physical and non-physical networks (Castells 1996).

Staying competitive in the global economy means fostering innovation and adapting new knowledge sources (Bathelt and Glückler 2011). Agglomeration economies enable and improve these processes through shared labor markets or spontaneous knowledge spillovers due to spatial proximity between firms (Combes and Gobillon 2015). At the same time, firms are increasingly embedded in functional networks fomed by cities and firms. In these networks, so-called “urban network externalities” (Capello 2000) arise. Here, cities are able to “borrow” (Camagni et al. 2016), exploit, or partially replace local agglomeration functions from other places, even ones that are not in physical proximity to each other (Capello 2000; Burger and Meijers 2016). This interplay of local externalities, through agglomeration economies, and connectivity, through network economies, plays a crucial role in the knowledge creation process and economic growth of a region (Bathelt et al. 2004; van Meeteren et al. 2016). Although various location factors such as universities, a high local GDP, international airports, or corporate taxes have been identified as beneficial for attracting firms, they are not equally important to all industries (e.g., Zandiatashbar and Hamidi 2018; Adler and Florida 2020; Sigler et al. 2020; Chong and Pan 2020; Wu et al. 2022).

The main goal of this paper is to explore how multi-branch, multi-location firms of advanced producer service (APS) firms and High-Tech industries shape and use space in Germany. Our assumption is that firms specifically choose their locations to not only increase their competitive edge but also add highly localized knowledge to their knowledge creation process. Furthermore, we expect that characteristics in space, such as the mode of transport and travel time between two places, have a significant influence on how and where firms operate their firm locations. Therefore, we assume that locations in multi-location multi-branch firm networks are not independent of each other but interconnected by specific location requirements, proximities, and accessibilities. This relational approach to observing firm networks requires the use of network analysis since the standard assumption of non-independence is violated, making statistical regression analysis infeasible.

In the literature, different network analysis approaches have been used to model location choices, including exponential random graph models (ERGMs) or stochastic actor-oriented models (SAOMs) (Liu et al. 2013b; Broekel et al. 2014; Block et al. 2019; Chong and Pan 2020). We opt for the ERGM approach for several reasons. First, SAOMs require specific actor-based assumptions, such as specific intra-firm considerations of locations that we do not have (Block et al. 2019). Additionally, SAOMs are not unproblematic when used in a spatial context (Broekel and Bednarz 2018). Second, we intend to understand why certain patterns of firm networks can be observed. The goal, thus, is to understand how these individual location decisions collectively shape the space we observe. Boschma and Frenken (2003) argue that, in addition to spatial concentrations, attention should be paid to the mechanisms by which knowledge is passed on, imitated, or adopted in firm networks. ERGMs provide us with the opportunity to examine these firm location patterns in Germany in more detail and go beyond the general description of location choices. Linking firm locations to the spatial characteristics of regions that are linked by transport infrastructure highlights the importance of accessibility and supra-local connectivity in firm networks. In this paper, we take the interlocking network model (INM) approach to conceptualize firm networks and relate it to local infrastructures and location factors. Taylor (2004) introduced the INM to approximate the underlying knowledge and information flows through a relationality between two places (Lüthi et al. 2018).

We base our analysis on a comprehensive dataset of firm locations, with locations of the 30 largest firms in 14 subsectors aggregated to knowledge bases in Germany and combine it with different modes of accessibility (rail, road, air). We chose Germany because it has a highly diversified economic structure, and the territory features an interesting urban system of cities and surrounding urbanized areas organized in a decentralized federalist structure. We show that the location choice of firms in a knowledge base, on the one hand, is strongly dependent on the type of sector firms are assigned to and, on the other hand, to some extent, a path-dependent development. However, distinct differences between the four bases are evident.

This paper is structured as follows. Section 2 briefly summarizes the theoretical background of knowledge networks and how knowledge gets created in space. Section 3 introduces the empirical approach, namely the exponential random graph model, as a way to model firm networks against infrastructural properties such as railway networks and introduces our datasets. Section 4 discusses the results of the employed models and their implications. Finally, Sect. 5 gives an outlook and concludes this study.

## Conceptual background and hypotheses

At the beginning of the 20th century, Alfred Weber and Marshall (1920) opened the theoretical discussion on the location choices of firms, agglomerations, and the advantages that arise with concentrations of firms, such as labor market pooling or economies of scale. Ever since these seminal works, an abundance of research has been put into the question of where and why firms locate, co-locate, or cluster. Moreover, the growing interest in the economic activities of multinational firms (e.g., Friedmann 1986; Castells 1996; Sassen 2001; Taylor 2004), more specifically in the context of the knowledge economy, altered the hitherto prevailing state of research on agglomeration economies (Bathelt and Glückler 2011). Here, local markets and transnational networks of firm locations are equally important to access even the most localized knowledge. However, separating the locality and globality of agglomerations in firm networks is not straightforward, as multinational firms usually use both in their internal operational processes.

For this reason, van Meeteren et al. (2016) propose an interpretation as a complementary continuum in which agglomeration processes operate primarily at the local level, but network effects predominate with increasing distance. We argue specifically that localization economies decrease with distance. These are then complemented by network economies, where distance is negligible.

Evolutionary economic geography started to unpack these firm spatial concentration processes quite differently by studying where and why firms (re-)locate or exit certain regions (Boschma and Frenken 2018). For example, do regions grow through spinoffs of parent companies, as Boschma and Frenken (2003; 2007) showed when they analyzed internal company routines? Or do they grow because of regional specialization economies, as Hidalgo et al. (2007) present in their work on “product space”? We argue that both are equally true in a multi-branch, multi-location knowledge economy.

Firms in well-developed economies seek qualified personnel—which is also the case in the knowledge economy (Storper and Scott 2009; Bathelt and Glückler 2011). However, specifically in these advanced economies, these talent pools are unevenly concentrated in space, so firms actively (re-)locate in such regions (Adler and Florida 2020). Bathelt and Cohendet (2014) summarize that at the core of the knowledge creation process, firms are embedded in local ecosystems to interact with other (in-)formal organizations but are integrated into global production networks. Especially large multinational organizations profit from network economies as they have the resources to access local tacit knowledge globally (Howells 2002; Simmie 2003; Bathelt and Glückler 2011).

This leads to an increasing concentration of economic activity, which can also be observed in Germany, where only a few cities and urban regions are gaining in importance (Krätke 2007; Brunow et al. 2020). Knowledge-intensive firms, and here especially APS firms, are widely regarded as important drivers of this formation process as they seek and incorporate new sources of knowledge to remain competitive in the market (Castells 1996; Sassen 2001; Brunow et al. 2020). Additionally, research on the knowledge economy system points to the fact that firms in High-Tech industries also create worldwide office networks. Some authors highlight their even more globalized firm network structure compared to APS firms (Lüthi et al. 2018).

Numerous works of literature around the Globalization and World City (GaWC) Research Network track the emergence of such knowledge-based firms or industries in many parts of the world, studying firm locations and economic developments alike. The GaWC uses multinational and multi-branch firms of the APS sector to statistically determine the relations between regions in a globalized world (Taylor 2004). Bettencourt (2019) follows a different path by presenting a framework of how city networks in total are formed—through social, economic, and infrastructure networks. The author argues that city networks are a complex construct of historical and evolutionary elements that should be primarily studied through socioeconomic connectivity since this interaction is how historically cities developed. While focusing on the location patterns of such firms, several studies found different location choices among APS sectors at the local city scale. (e.g., Vandermotten and Roelandts 2006; Bassens et al. 2020; Chong and Pan 2020; Waiengnier et al. 2020). Waiengnier et al.’s (2020) study on Brussels, for example, shows that the location preference, and therefore the concentration of firms, depends on the size of the firm and the service they provide. In addition, the authors found that knowledge-intensive firms value centrality but, at the same time, various aspects within such agglomerations, such as market accessibility or path dependency. Chong and Pan (2020) confirm these findings on location choices by employing an ERG model to study how APS firms form a city network between different cities in China. The authors further emphasize the importance of an efficient rapid transportation network to foster network effects.

Still, several shortcomings emerge regarding these studies: they focus primarily on the agglomeration effects between APS firms or specifically study one city or region and therefore are of limited comparability. We interpret space differently by combining positional data, thus the features of regions, with the relational characteristics of both firm networks and how regions are actually connected through roads, air routes, and rail networks.

With a focus on High-Tech-oriented firms, Boschma and Wenting (2007) and Harris et al. (2019) found that competition and related activities are more important for the manufacturing industry in Britain than densely populated regions or being located in a major city. However, as of now, and given that we focus on the relationship between positional and network data, not many comparisons of APS and High-Tech firms have been carried out. In this paper, we focus not only on APS firms but on the full spectrum of knowledge-intensive firms and follow Lüthi et al. (2011) to define the knowledge economy as “that part of the economy in which highly specialized knowledge and skills are strategically combined from different parts of the value chain in order to create innovations and to sustain competitive advantage.”

However, since the knowledge economy is not a homogeneous construct, it is crucial to differentiate between the various sectors to identify its dynamics more precisely (Kronenberg and Volgmann 2014). In order to quantify this complex spectrum of the knowledge economy, we make use of the knowledge base typology by Asheim and Gertler (2006). The authors propose a classification into three knowledge bases. Synthetic and symbolic knowledge incorporates a strong tacit body, while in analytical knowledge, codifiability is the dominant form (Asheim and Hansen 2009). Furthermore, these three knowledge bases have different sensitivities to geographic distance (Asheim et al. 2011).

Firms with an analytical base focus on activities with a strong scientific component, such as biotechnology or information technology (Asheim and Gertler 2006). Due to the aforementioned codifiability of analytical knowledge, it is less sensitive to distance, and the transfer can be utilized more easily across space (Storper and Venables 2004; Tranos 2020). Firms with a strong focus on symbolic knowledge, such as design, media, or advertising firms, depend on physical proximity; social interaction and networking are key elements in promoting the necessary knowledge transfer (Asheim and Gertler 2006). Therefore, we often find industrial clustering of firms with a symbolic knowledge base (Tranos 2020). Lastly, firms with a synthetic knowledge base focus on combining and applying existing knowledge. Such firms rely more on tacit knowledge and, therefore, geographic distance due to the nature of the application of knowledge and interaction, or learning by doing (Asheim and Hansen 2009). Zhao et al. (2017) applied the knowledge base typology to analyze the location choices of knowledge workers. The authors identified four economic groups: symbolic APS, synthetic APS, synthetic High-Tech, and analytical High-Tech. The authors show that different location preference patterns are evident depending on the knowledge base. The authors further find that workers with a symbolic knowledge base show the greatest preference for urban locations, followed by APS workers with a synthetic knowledge base.

On the other hand, workers in High-Tech firms generally show less preference for urban locations. These results are in line with the literature above. However, contrary to that, the authors also identify the possible preference of synthetic APS workers for peripheral areas. Therefore, we expect different location choices for analytical and synthetic High-Tech firms, which consist of manufacturing and research-oriented firms. Furthermore, Van Oort (2004) and Raspe and van Oort (2011) found that firms profit less from their proximity to universities than from being centrally located. Building on this fundament of literature, we hypothesize that analytical and synthetic High-Tech firms have a lower propensity for urban locations than synthetic and symbolic APS firms (H1).

Knowledge-based firms rely on local infrastructural amenities. The literature agrees that universities and co-operating or industry-specific R&D institutes are key factors for firms to locate in a specific place (e.g., Audretsch and Stephan 1996; Simmie 2003; Florida et al. 2015). For example, Arant et al. (2019) show that collaborations between firms and universities can be fruitful for the innovation process, as it gives them access to new knowledge. In addition, Roesler and Broekel (2017) identify universities as nodes in a network that knowledge-intensive firms can access. Besides these beneficial collaborations between universities and firms, the literature shows that the societal, cultural, and creative image, but also the presence of recreational amenities, attracts highly skilled workers and thus knowledge-based firms (e.g., Trip 2007; Carlino and Saiz 2019; Wu et al. 2022).

Zandiatashbar and Hamidi (2018) found that the walkability and transit service quality of a place have a positive impact on attracting knowledge-based firms, thus positively influencing innovation. Earlier, Karlsson and Andersson (2004) identified a close link between accessibility and the performance of a region. The authors argue that travel time is a more appropriate measure of accessibility than physical distance. In contrast, de Bok and van Oort (2011) found no significant results for firms in the manufacturing sector and suspect a trade-off between accessibility and the cost of rent since these firms require more space to do business. Coscia et al. (2020) found that business trips decline with travel-time distance, hence why APS firms heavily depend on transportation infrastructures to facilitate face-to-face business activities. For these firms, airports are seen as nodes in a global network of firm locations. Neal (2012) and Liu et al. (2013a) highlight airports as infrastructures that stimulate business activities and air passenger networks. Further, Conventz and Thierstein (2014) identify airports as hubs for events and conferences, making them also useful for short business trips.

Particularly in the Asian and European context, (high-speed) rail infrastructure complements this relationality. Wenner and Thierstein (2021a, b) show the role of high-speed rail for regional accessibility, replacing air traffic on an increasing number of (inter-)national routes as the fastest mode, and the role of certain high-speed rail stations for urban development, including locational decisions of firms. Zhao et al. (2017) found that both symbolic and synthetic APS knowledge workers tend to utilize public transport or active modes to commute more than workers in High-Tech firms.

Regarding the transport infrastructure, Krenz (2019) identified that road accessibility and short travel times are important factors for German High-Tech firms. However, Zhao et al. (2017) highlight that synthetic High-Tech workers show a higher preference for commuting by car—and therefore road accessibility—than analytical High-Tech workers. Therefore, we assume that there is a measurable difference in how firms value different modes of accessibility—road, air, and train—to best leverage their individual networks for the exchange of information and, eventually, the creation of knowledge. This leads to the second hypothesis: We hypothesize that APS firms, in general, demonstrate a stronger preference for rail and air accessibility, while among all High-Tech firms, those with a synthetic knowledge base show a stronger preference for road connectivity (H2).

Before we present our methodology below, we present our approach to the underlying firm location decision process that is ultimately reflected in this study’s dataset. We think this is an important step in understanding how space is constructed and shaped. As stated before, much research (e.g., Smętkowski et al. 2021) focuses on location factors within a city or a region. Therefore, in these studies, the focus lies on a small set of firms that choose locations based on given location factors or amenities, e.g., qualified personnel and favorable infrastructure, to exploit local knowledge sources or competitive markets. However, when focusing on firm networks rather than individual firm locations, a different approach is needed. Firms, especially knowledge-based firms, constantly re-evaluate whether the given location in their firm network still meets their internal requirements and react accordingly by relocating or adapting their network. Infrastructure, for example, must be maintained or built so the regions themselves, through public and private investment, become players competing globally to attract and retain firms in a region (Boschma and Frenken 2018; Bettencourt 2019). Moreover, a firm’s location choice is influenced by the workers’ requirements, e.g., whether proximity to a train station is important or not (e.g., Zhao et al. 2017)—hence, firms may move individual locations based on changing employee demands. Therefore, when focusing on particular firm locations, we argue that such locations can be compared to nodes in the firm-location network shaped by local specifications and workers’ location preferences.

In this study, however, we go a step further by aggregating individual firm networks to a much broader firm-location network. Here, locations—their specific amenities and infrastructures—emerge as central not just to one firm but to many of them, making them key actors in the network. Thus, by overlaying dozens of firm networks, we are able to not only draw conclusions about the general behavior of knowledge-intensive firms in space but also demonstrate a hierarchy of areas where they locate.

## Methodology and data

### Exponential random graph model

In this section, we discuss the setup and function of the exponential random graph model (ERGM), which we use to detect a distinct spatial pattern in our firm dataset. In short, ERGMs are statistical tools to describe the likelihood of given network structures by modeling an infinite number of possible networks and comparing these to the global structure of a network (Hunter et al. 2008). However, ERGMs are computationally intensive; hence we opted for Krivitsky et al.’s (2021) estimation approach that relies on a central Markov chain Monte Carlo (MCMC) algorithm for estimation and simulation along with a maximum pseudo-likelihood estimator for the results. In this study, we aim to explain the given network structures of each subsector in the knowledge economy by modeling a random network model using the ERGM and additional node-level data.

ERGMs are similar to regression models, except that ERGMs do not imply that all parameters are statistically independent (Van Der Pol 2017). The underlying network used in our ERGM is the interlocking network model (INM), as presented later in this section. However, the conventional ERGM to be estimated is limited to binary data, meaning that it can only estimate the probability of a tie between two regions on a binary level (Krivitsky 2012). As this methodology is discussed in detail by Hunter et al. (2008), Krivitsky (2012), and Krivitsky et al. (2021), we will present a parsimonious summary. We use functional urban areas (FUAs) as our spatial scale, which we briefly introduce in 3.2.3.

Transforming network data into binary results in a loss of information, specifically in the categorization of firm locations, according to Taylor (2004). We, therefore, use Neal et al.’s (2021a) backbone approach to dichotomize our valued INM dataset into binary space. We extracted the backbone of our dataset using the fixed degree sequence model (FDSM) with a two-tailed α of 0.05. We used this alpha for three of the four knowledge bases as we found this to be the best compromise between a good fit and preserving network details of less “meaningful” (Neal et al. 2021a) connections. For the knowledge base synthetic APS, we chose a stricter α of 0.01 to improve the fit. For the statistical and mathematical background of this approach, we refer to the relevant literature by the authors (Neal et al. 2021a, b). Lastly, we follow Duxbury’s (2021) approach to check for multicollinearity in ERGMs and did not detect any. The authors will provide the results upon request.

In general, an ERGM models the probability distribution over all possible networks ($\forall x\in \mathrm{X }$). It takes the form of Eq. 1:

1 $$Pr\left(X=x|\theta \right)=\frac{\mathit{\exp }\left(\theta ^{T}s\left(x\right)\right)}{k\left(\theta \right)}\forall x\in X$$

Where *x* is the given (observed) INM network structure, *X* is the random (modeled) network modeled by the ERGM, *θ* is a vector of weights, model parameters *s*(*x*) is a vector of the network parameters or exogenous variables, and *k*(*θ*) is a normalizing constant. In our study, we used two different types of exogenous variables: Co-variables that describe each FUA on the nodal level in more detail, i.e., inhabitants or whether an FUA features an institution of higher education. On the other side, we used co-variables that describe the relationship between two FUAs, in the form of physical distances, travel times, railways, or connecting flights. We will present the results of the ERG model in more detail in the next section.

### Data and model specification

#### Firm data

Our dataset is formed by firms in the German knowledge economy and their respective branch locations in Germany. In identifying knowledge-intensive firms, we follow the classification by Legler and Frietsch (2006) and its updated version, Gehrke et al. (2013), based on the German Classification of Economic Activities (Wirtschaftszweige 2008). Even though this taxonomy has its shortcomings, such as the possibility that, over time, firms may have shifted operations to different economic activities, we still think this classification is robust enough since it focuses on research-intensive industries, i.e., firms that use, reuse, or create new knowledge. We further aggregated knowledge-intensive classes of economic activities into groups with a similar activity-related context. Here, we followed Zhao et al. (2017) and formed four groups, analytical High-Tech (AnH), synthetic High-Tech (SyntH), synthetic APS (SyntA), and symbolic APS (SymbA). The following subsectors were formed: In synthetic APS (SyntA), we included the sectors accounting, banking and finance, insurance, law, management and IT consulting, information and communication services, and third- and fourth-party logistics. In symbolic APS (SymbA), we grouped the advertising, media, design, architecture, and engineering sectors. In the knowledge base of synthetic High-Tech industries (SyntH), we defined the following subsectors: mechanical engineering, computer hardware, electronics, telecommunications, and vehicle construction. The last group analytical High-Tech (AnH), includes the chemical & pharmaceutical and medical & optical instruments sub-sectors. These industries were chosen because of their investment in research and development activities and a high share of highly-skilled labor (Legler and Frietsch 2006). We created the firm database using manual and semi-automatic web-scraping methods of the online self-presentations of the 30 largest firms by employees in Germany in each group. We opted for the cut off at 30 since we aimed to cover firms that are located all over Germany and argue that this number is sufficient to make well-founded statements about the German knowledge economy. We extracted and geo-referenced 17,786 unique firm locations.

#### Interlock connectivity

The underlying two-mode network uses the interlocking network model (INM) methodology. Taylor (2004) conceptualized this methodology to measure firm and city networks globally. The INM has been used to rank cities according to their position as global centers of command and control in various contexts, both economically and politically (e.g., Derudder and Taylor 2016). In this paper, we take this approach to conceptualize firm networks and relate them to local infrastructures and spatial amenities. For each firm, all office locations were assigned a *service value* between 5 and 0 according to the presence of the firm within the FUA. Although this valuation is not required in the ERG model, it serves as a guide to understanding where firms are located or headquartered.

An important parameter in the analysis of the firm network is the sum of all firms’ service values (*j*) per FUA (*a,b*), i.e., the number of firms per FUA weighted by the importance of their regional presence (*v*). The INM approach then infers the strength of the relation (for example, the quantity of information exchanged) between two firm sites by multiplying their (firm-internal) service values, which we call the *elemental interlock* (*r*_*abj*_). A generalized score for an FUA-to-FUA relationship is then derived by summarising the strengths of all firm relations between the FUAs, called the *FUA interlock*. By again summarising the strength of all FUA interlocks to and from an FUA, a nodal value representing the embeddedness of an FUA in national firm networks can be obtained, which we will call *interlock connectivity*.

2 $$\sum _{j}r_{\mathrm{abj}}:=v_{aj}v_{bj}$$

The resulting network is a one-mode network as we study the relation of a subset of firms with locations in specific FUAs and not of individual firms on the node level directly. We, therefore, calculate the one-mode network separately for all four knowledge bases. These networks are then transformed in binary space using Neal et al.’s (2021a, b) backbone model to get them to the appropriate form for the ERGM; see above.

In the following two sub-sections, we separately introduce all node and dyad level variables we used.

#### Node-level data

In both sections, to keep matters short, we introduce abbreviations in paratheses. We mapped and binary coded all public institutions of higher education (universities and universities of applied sciences) (INST) and airports (AIRPORT) in Germany.

This spatial base unit of our analysis is based on the urban centers identified in the ESPON 111 project for Germany and nearby regions (ESPON 2004). The project has defined urban centers and their hinterlands (so-called PUSH areas—potential urban strategic horizon) using a set of functional-spatial criteria such as in-commuting patterns. This results in a list of 186 FUAs for Germany. Importantly, the PUSH areas were defined regardless of national boundaries, which means that some FUAs extend into neighboring countries, while other border areas of Germany are not covered by an FUA.

Since we assume different locational preferences depending on the economic output or broader sector we classified each firm in, we made use of the spatial classification published by the Federal Institute for Research on Building, Urban Affairs and Spatial Development (BBSR). Each municipality is categorized according to its population density into one of the three following categories: urban areas (1; URBAN), semi-urban areas (2; SEMI-URBAN), and rural areas (3; RURAL) (BBSR 2019).

Additionally, we follow the BBSRs categorization of cities according to their population size. A *Großstadt* (> 100 k) features at least 100,000 inhabitants, a *Mittelstadt* (< 100 k) between 20,000 and 100,000 inhabitants (source: BBSR). Since there is at least one *Kleinstadt* (less than 20,000 inhabitants) in each FUA, we opted for > 100 k and > 100 k only. Similar to the threefold BBSR classification, we counted and transformed the classification to fit our FUA dataset. (Table 1).

**Table 1:** Classification of functional urban areas in Germany, applying BBSR categorization (source: BBSR)

	Count	Average population over all cities	Average employment over all cities	Average size
*Großstädte* *(>* *100* *k)*	68	757,235	413,170	1228 ha
*Mittelstädte* *(<* *100* *k)*	118	266,081	132,378	1954 ha

Lastly, we added the German metropolitan regions (METROP) as a dummy variable to our dataset. In Germany, 11 metropolitan regions are identified, usually consisting of at least one *Großstadt* and several smaller regions or districts. Metropolitan regions in Germany are thought to be drivers of economic, social, and cultural development (Zimmermann et al. 2020).

Since each FUA is computed using NUTS regions on the smallest statistical entity, the spatial unit used in Germany is the municipality. Each FUA may consist of one or more statistical entities, forming the aggregated FUA. As mentioned above, FUAs bordering neighboring countries cover municipalities outside Germany. In these cases, we used data provided by Eurostat on the level of local administrative units (LAU).

A similar approach was chosen to gather employment data. Our dataset for the German Districts comprises data from the Statistical Office of Germany for the neighboring European countries from Eurostat. As data is not collected for the countries Liechtenstein and Switzerland, we opted for official data provided by the Statistical Offices of both countries.

#### Dyad-level data

We calculated several proximity factors towards all other FUAs to measure transport-based accessibilities. First, we extracted and counted all direct connections by train between two FUAs (AMNT TRAIN) using the current online railway timetable of the German railway operator, Deutsche Bahn (DB 2020). Then, we calculated the *efficiency* of a train connection between two FUAs (PROX TRAIN). For this, we gathered the travel time by obtaining the time schedule of Deutsche Bahn for each central railway station in each FUA. Then, we used these travel times between two FUAs (*tt*_*train*_) and related them to the geodesic distance between two FUAs a and b (*dist*_*ab*_). This ratio represents the *efficiency* of a connection:

3 $$\textit{efficiency}_{\mathrm{rail}}=\frac{\textit{dist}_{ab}}{tt_{\mathrm{rail}}}$$

We also calculated the road distance for each FUA-to-FUA (PROX ROAD). Further, we also conducted a road-versus-rail network analysis by comparing the efficiency of the travel time in a train network against the road network (PROX RR). Again we calculated for each connection using the following term:

4 $$\textit{road versus rail}\left(RR\right)=\frac{tt_{\mathrm{rail}}}{tt_{\mathrm{road}}}-1$$

Here, *tt* is the travel time using either the road or rail network. Firms may choose a location to minimize time between two places and thus prefere different modes of transport. With this term, we study which network is the better option: The term is positive if the connection is faster using the road network and becomes negative if it is the rail network. Lastly, we collected the number of direct flights between each of the airports in Germany. Since not all FUAs in our dataset host an airport, the network of air flights is rather tight, forming the third type of proximity for our analysis (AMNT AIR).

PROX TRAIN suggests that with 2685 direct train connections (out of 35,156 possible connections), only a handful of FUAs are connected to all other FUAs in the network by rail. PROX AIR suggests an even smaller network of 23 direct connections between FUAs. PROX RR, the ratio of travel times between two FUAs, is slightly negative, suggesting a faster connection using the train network over the road network for only 16.4% of the road network represents a significant advantage. The estimation method defaults to the Monte Carlo maximum likelihood estimation (MCMLE) (Krivitsky et al. 2021). The findings are presented and discussed below.

## Findings and discussion

### Model validation and fit

The network used in our analyses in the ERGM takes the form of a two-mode dataset and describes the relation between two cities through a sum of links for each individual firm in each subsector. We use R packages to implement the ERGM developed by Hunter et al. (2008), Krivitsky (2012), and Krivitsky et al. (2021).

It is important to stress some methodological issues that we had to overcome. First, we must acknowledge that we have only taken the 30 most important firms of each subsector of the German knowledge economy under consideration. Therefore, our firm sample is not a statistically independent and identically distributed random sample of firms. It was never the intention to do so, and we, therefore, do not draw any conclusion on the population of the German knowledge economy as a whole.

We followed Statnet (2021) and tested the goodness-of-fit for each model and assessed the degree distribution. Since each subsector is one model, the results are in the appendix. One should recall that we used Neal et al.’s (2021a, b) FSDM approach to extract the backbone of each network. We, therefore, compare the backbone of a network with its modeled counterpart. We discuss the empirical results of the ERG models below.

### Results and discussion of the ERG models

We begin by presenting the general effects of our data on the model. The results of each model for all sectors are presented and compared to validate our hypotheses later. Our base for each variable is a peripheral region (RURAL) that has no institution of higher education (INST = 0) and is not part of a German metropolitan region (METROP = 0). We separate the results by hypothesis and present both APS and High-Tech subsectors. The full results for all subsectors are presented in the appendix. Each column represents a separate individual model. The rows indicate the effect of each parameter in the form of coefficients.

#### Principle effects

Across most subsectors, the presence of institutions of higher education (INST) in an FUA has a significant positive influence in forming a link between two FUAs. This comes as no surprise since economic activity happens in more densely populated areas, and higher education institutions in Germany are usually located in core city areas. This is mostly true at the < 0.05 significance level for subsectors in either APS or High-Tech. Interestingly, being located in a metropolitan region (METROP) has a negative impact on several branches of both High-Tech and APS. We suspect this is due to the fact that our base model suggests a location that is classified as peripheral but at the same time is not in a metropolitan region. A peripheral region in a metropolitan region could be subject to drainage of economic activity from the region’s core. Similarly, the number of direct connections by train between two FUAs (AMNT TRAIN) has a significant positive influence. These assumptions are only to be considered with all other variables held constant. In the following sections, we will discuss the results for each hypothesis.

#### Hypothesis (1): Analytical and synthetic High-Tech firms are less centrally located

The results of the models partly yield evidence that High-Tech firms are located in less central locations. We do generally assume that a firm is more centrally located if the estimates for an FUA, the number of *Grossstädte* or location in an urban area, yield significantly positive results. For this hypothesis, we look at the parameters URBAN (urban area), SEMI-URBAN (semi-urban area), METROP (the FUA is part of a metropolitan region), > 100 k, and < 100 k (whether the FUA hosts a *Grossstadt* or *Mittelstadt*). Starting with the parameter METROP, we identify an interesting pattern, as it is negatively significant for both High-Tech knowledge bases. However, we must keep in mind that our base model is a rural FUA without an institution of higher education and not being part of a metropolitan region. Here, we argue that an FUA in a metropolitan region is not enough to attract firms operating in the High-Tech knowledge bases. Regarding the estimates for > 100 k and < 100 k, we can summarize the following observations: Firms in synthetic High-Tech seem to prefer *Mittelstädte* and not *Grossstädte*, both estimates are positive yet not significant. Further, analytical-based firms are significantly positive on FUAs with cities < 100 K inhabitants and therefore seem to prefer higher populated urban areas (URBAN). This contradicts our hypothesis at first. However, with regards to the literature we discussed earlier (e.g., Asheim and Gertler 2006), we suspect that firms locate in such areas, and span firm networks between big cities. This showcases an interesting finding as we clearly identify different location patterns for High-Tech firms. Recall that in our dataset, synthetic-based (SyntH) firms are, for example, automotive manufacturers and suppliers, electronics or mechanical engineering firms, while analytical High-Tech (AnH) firms are associated, for example, with the chemical & pharmaceutical industry. Similar to Krenz (2019), we conclude that German manufacturing firms seem to prefer locations in agglomeration areas, possibly in smaller *Grossstädte* (< 100 K) or near *Mittelstädte*, but with good infrastructure and accessibility for workers.

When focusing on APS knowledge bases, both analytical and symbolic knowledge-based firms return mixed results. Synthetic APS (SyntA) returns significantly positive results in semi-urban parts of Germany and significantly negative results for German *Grossstädte* (> 100 K). We argue that this is due to the well-developed networks of banking & finance and insurance firms, which need to be close to customers and therefore have a strong presence in all parts of Germany. Both synthetic (SyntH; SyntA) and symbolic (SymbA) knowledge bases return significantly positive results for the variable INST—highlighting that an institution of higher education in a city is a major factor in the location decision.

Regarding our first hypothesis, we summarize that High-Tech firms in our dataset show evidence of expected location patterns. Firms with an analytical knowledge base (AnH) are significant in parameters that we associate with being central. Synthetic High-Tech (SyntH) firms seem to favor less central FUAs to set up firm networks, with an urban structure (URBAN) being significantly positive. Contrary to our assumption, we do not witness synthetic APS (SyntA) firms as the most centrally concentrated, with no significant outcomes in the variables under consideration. We suspect this is due to the well-developed network of firm locations in the banking & finance and insurance subsectors which mirrors the population distribution, thus making the spatial patterns appear random. In total, we can only partly confirm our first hypothesis by a combination of > 100 K, < 100 K, and URBAN.

#### Hypothesis (2): APS firms depend more on railway and airport accessibility while synthetic-based High-Tech firms prefer road accessibility

Starting with the dyad estimates, the parameter PROX TRAIN measures the travel time by train between two FUAs. It is significantly negative for synthetic APS (SyntA) firms. The coefficient focusing on the number of train connections between two FUAs, AMNT TRAIN, identifies another pattern as both synthetic knowledge bases (SyntA; SynthH) and symbolic APS (SymbA) are significantly positive here. We suspect that short travel times by train may be an indication of industrial clustering, as showcased by Broekel and Bednarz (2018). We further deduce that the distance by train between two FUAs may have an influence on the formation of a tie: As the distance between two places increases, the probability of a connection decreases.

The parameter AIRPORT is significantly positive in synthetic High-Tech (SyntH). Again, we interpret this with the smaller-sized, less extensive firm networks in all knowledge bases but synthetic APS (SyntA) (Tether 2002). For firms with a symbolic (SymbA) or synthetic (SyntH) knowledge base, the results show evidence, in line with previous results (e.g., Thierstein and Conventz 2014), that these firms interact less on a regional and more on a national and global level as firms in both knowledge bases seem to make use of airport connections as both are also significantly positive for AMNT AIR—the number of direct flights between two FUAs.

Regarding the preference for road accessibility, we study the estimated parameters PROX RR and PROX ROAD. Recall that the interpretation of PROX RR is the ratio of travel times for road and railway connections, meaning that a positive outcome in PROX RR signals a more important, and thus faster, road network connection. Both synthetic-oriented knowledge bases feature a negative but statistically not significant estimate. The parameter PROX ROAD is positive for analytical firms, negative for synthetic-based High-Tech firms, and significantly negative for synthetic APS (SyntA) firms. Even though the parameter is not significant for High-Tech firms, it may be a possible indication of the preference for road accessibility to transport goods, as shown by Krenz (2019).

In any case, we cannot confirm this hypothesis as there is no clear distinction for airport accessibility, as only firms in synthetic High-Tech (SyntH) yield positive results here. We suspect that one major influence is the network structure itself of the firms in our dataset. For example, in synthetic APS, banking firms host dense networks with many subsidiaries, while in synthetic High-Tech, the value creation is concentrated at only a small number of locations in Germany. Lastly, even though the direction of road accessibility confirms our initial hypothesis, the estimates are not statistically significant, hence why we cannot confirm the hypothesis and conclude that further research is needed.

## Conclusion and research outlook

With this study, we expand the theoretical debate on the locational behavior of firms in the knowledge economy. We do this by examining the influence of physical accessibility on firm locations using exponential random graph models (ERGM). ERGMs are used in social network analysis. However, since ERGMs allow us to study parameters affecting the formation of ties in a network, it offers a valuable research path to study firm networks. Since we utilize the renowned interlocking network model (INM) to map firm networks, first introduced by Taylor (2004), using an ERGM seems feasible.

Our data is based on FUAs and our results confirm previous findings on firm locations and accessibility to transport infrastructures, even though those other studies were conducted on individual city spaces (De Bok and Van Oort 2011; Smętkowski et al. 2021; Wu et al. 2022). In this study, we departed from dividing the knowledge economy into the two main segments of APS and High-Tech. Instead, we followed Zhao et al. (2017) by assigning the firms with their respective characters of economic activities to one of the three different knowledge bases defined by Asheim and Gertler (2006) and Asheim and Hansen (2009). Thus, we use a typology with four types of economic activities: symbolic APS (SymbA), synthetic High-Tech (SyntH), and analytical High-Tech (AnH).

The results of the ERGM confirm the descriptions of knowledge bases: Firms with an analytical knowledge base have a significant location preference in FUAs with a dense population and German *Grossstädte*, highlighting their less geography-sensitive firm networks (Asheim and Gertler 2006). On the other hand, firms with a symbolic knowledge base depend on face-to-face social interaction also favor German *Grossstädte*. We assume any type of *Grossstadt* is sufficient (both > 100 K and < 100 K). For synthetic APS (SyntA), our results indicate that firms are distributed across German FUAs in patterns that we would also expect to find at random. In the synthetic knowledge base, which also includes fourth-party logistics firms, our results indicate a preference for the road network and airports over the rail network while being located in semi-rural regions—a possible indication of a faster or more convenient connection. Contrary to our expectations, symbolic APS (SymbA), not synthetic APS (SyntA), firms favor locations with short airport accessibility. Again, we suspect this result from the dense network of firm locations in the banking and insurance sector here.

We argue that current developments are following a common theme, as the once clear-cut dichotomy of High-Tech firms as the sole producers of goods and APS firms as suppliers of services is diluting more and more. Whether in the automotive industry, the biochemical industry, or consulting branches, more and more firms are integrating in-house consulting services or data analytics facilities to leverage new technologies, such as artificial intelligence, to support standardized processes. Such adaptions require similar knowledge resources that can only be found in certain regions or cities (Isaksen et al. 2020). This is leading to an increasing blurring of sectoral categorizations of firms in industries, as, for example, we used in this study.

Based on these findings, we state that grouping firms of the knowledge economy into only two broad groups that distinguish manufacturing from services—that is, APS and High-Tech—does not suffice anymore to identify distinct spatial location patterns comprehensively. These are strongly determined by the population of an urban area, functions, local features such as universities, accessibility, and preferred mode of transport. Therefore, we propose to discriminate the knowledge-intensive economic activities into four knowledge bases: analytical High-Tech, synthetic High-Tech, synthetic APS, and symbolic APS (Zhao et al. 2017). Because we chose to study only dyadic independence models for each network of knowledge base, there remains significant research prospect. We argue that dependent models will provide further insights into location choices—though cross-sectional dependencies are another promising area of research. Nonetheless, our approach offers an entry point into a methodology of using ERG models that goes beyond (independent) descriptive point data: large-scale network data from firm networks can be used to simultaneously examine where multi-branch firms locate, what type of location is preferred and how proximity to transport infrastructure is valued differently across sectors.

Policymakers seeking to emulate successful knowledge-based economic environments, such as Silicon Valley, should therefore carefully reassess their regional and local settings. We summarize that our results indicate the extent a region can attract firms of certain knowledge bases but also what infrastructure is actually required—or needs ot be in place in a region—in order to attract the *‘right’* firms.

There are some shortcomings to our approach. First, some networks in our dataset are too dense in form to be directly used. We, therefore, applied Neal et al.’s (2021a, b) backbone model to transform the INM into binary space. The backbone model uses significance levels below which less important connections are omitted and modifies the network depending on the selected alpha. Second, the use of FUAs as a spatial unit of analysis, composed of an urban center with its surrounding area, could also be broken down to finer levels of analysis; however, the use of such levels also increases the computational complexity. Lastly, a longitudinal analysis is needed in order to explore the changing locational patterns of current firm locations. It also needs to be stressed that in this paper, we focus on the applicability of ERGMs on a dataset of firm networks and location features in an explorative way; hence why we did not do any reverse-causality test of transport infrastructures, spatial amenities, and economic activity in space. Beyond these methodological limitations, one should not forget that we focused on location choices in Germany. These findings may be transferable for other central European countries with similar settlement and infrastructure patterns that originate in a more decentralized-federal organisation of the state. However, further reseach is certainly needed for other highly industrialised or emerging economies. We believe that further exploration of other aspects that may influence the location choice, such as the price per square meter of commercial space, could provide even more precise findings.

Looking ahead, an open question for future research comes to the fore: If knowledge-intensive firms increasingly seek specific occupational skills such as computational and digital high-level literacy, where in space—in which locations and on which spatial scale—do they source it from? Eventually, such research would contribute to the resurfacing of the long-standing challenge of understanding spatial structural change: At what point in time and where in space do firms follow knowledge workers, or at what point in time and where in space is it the other way around?

## Figures and Tables

**Fig. 1:**
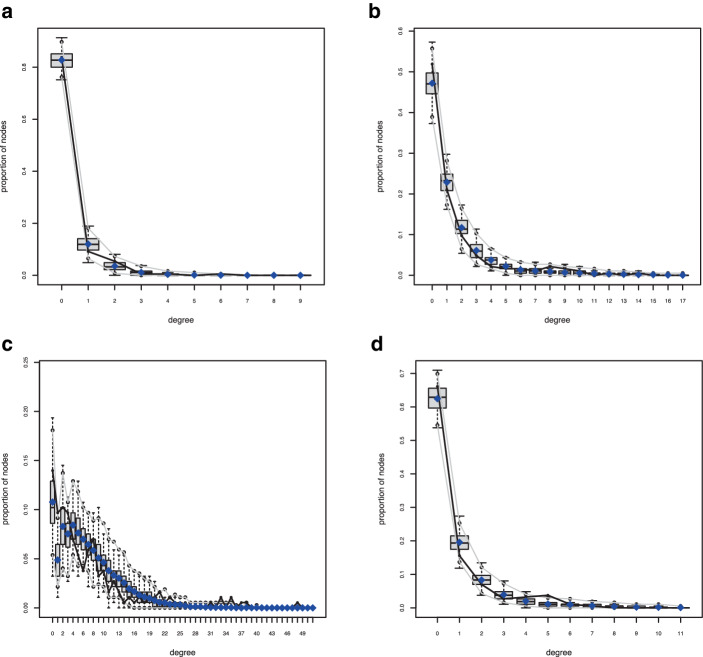
Degree distribution of all models. **a** Analytical High-Tech, **b** Synthetic High-Tech, **c** Synthetic APS, **d** Symbolic APS

## Appendix

**Table 2:** Results of the ERGM of all models

Variables	Analytical High-Tech	Synthetic High-Tech	Synthetic APS	Symbolic APS
*Node Level Data*				
Base (RURAL, METROP = 0, INST = 0)	−7.657403 ***	−6.1359503 ***	−4.5706791 ***	−7.3835881 ***
	(2.075337)	(1.0213286)	(0.3570418)	(1.4934690)
GWESP	2.516367 ***	1.5299296 ***	2.2670165 ***	1.7843039 ***
	(0.327809)	(0.1527263)	(0.1285344)	(0.2089514)
INST	0.427989	0.4886928 **	0.2129041 ***	0.8960853 ***
	(0.301600)	(0.1490778)	(0.0396019)	(0.2342771)
METROP	−0.715169 *	−0.2569601	0.0449865	−0.2541782
	(0.308217)	(0.1331800)	(0.0366146)	(0.1869199)
AIRPORT	0.006425	0.4204711 **	−0.0001216	0.2211411
	(0.359578)	(0.1513986)	(0.0663166)	(0.2275915)
< 100 K	0.285304 *	0.0260576	0.0327642	0.1084815
	(0.132709)	(0.0812587)	(0.0257093)	(0.1025858)
> 100 K	0.728396	0.2733456	−0.6088574 ***	0.4138008
	(0.538650)	(0.2293358)	(0.1344232)	(0.3295138)
MITTEL	0.052254	0.0203635	0.0125618	−0.0226790
	(0.044606)	(0.0145306)	(0.0073100)	(0.0235400)
SEMI-URBAN	0.023386	0.2638678	0.1362346 **	−0.0212625
	(0.422577)	(0.2412140)	(0.0513599)	(0.3004678)
URBAN	−0.091518	0.4142811	−0.0179168	0.1543739
	(0.044606)	(0.0145306)	(0.0073100)	(0.0235400)
*Dyad Level Data*				
AMNT TRAIN	0.006588	0.0054730	0.0155278 ***	0.0077997 *
	(0.005572)	(0.2412140)	(0.0513599)	(0.3004678)
PROX RR	0.001214	−0.0445525	−0.0311974	−0.0088865
	(0.105548)	(0.2207886)	(0.0498700)	(0.2710306)
PROX TRAIN	−1.129091	0.0663890	−0.5831535 ***	−0.5887913
	(0.711540)	(0.0039750)	(0.0022247)	(0.0036643)
PROX ROAD	1.490212	−0.9644960	−1.0019936 **	0.7360066
	(2.164089)	(0.3462289)	(0.0294892)	(0.1543991)
AMNT AIR	−0.006120	0.0003264 **	0.0006995 ***	0.0005532 ***
	(0.022769)	(0.0001029)	(0.0001007)	(0.0001409)
AIC	333.4	1229	4580	725.9
BIC	449.5	1345	4697	842.2

All continuous predictors are mean-centered and scaled by 1 standard deviation. *** *p* < 0.001; ** *p* < 0.01; * *p* < 0.05;. *p* < 0.1

**Table 3:** Descriptive Statistics

*Regions classified according* *to BBSR*	Count	Distribution of airports	Distribution of institutions of higher education	Distribution of Metropolitan Regions
*1; Urban*	106	19	63	65
*2; Semi-Urban*	40	4	22	19
*3; Rural*	66	5	11	19
